# TRAP sequence mimicking placenta bipartita: A diagnostic challenge in prenatal imaging: Case report

**DOI:** 10.1016/j.ijscr.2025.112010

**Published:** 2025-10-06

**Authors:** Sofia Mchichou, Rim Laaboudi, Chayma Chakir, Soundous Amine, Samir Bargach, Fatima Elhassouni

**Affiliations:** Gynaecology-Obstetrics and High risk pregnancy Department, Maternity Souissi, University Hospital Center IBN SINA, University Mohammed V, Rabat, Morocco

**Keywords:** TRAP sequence, Acardiac twin, Fetal intervention, Monochorionic pregnancy

## Abstract

**Introduction:**

Twin Reversed Arterial Perfusion (TRAP) sequence is a rare complication of monochorionic twin pregnancies, where one twin without a functional heart is perfused retrogradely by the healthy “pump” twin. Early diagnosis by ultrasound and timely intervention, such as laser photocoagulation, can significantly improve the prognosis of the pump twin by reducing the risk of cardiac failure and preterm birth.

**Case report:**

A 33-year-old woman at term presented with decreased fetal movements and was diagnosed with intrauterine fetal demise and a placenta bipartita. Cesarean section for suspected chorioamnionitis revealed a stillborn fetus and an acardiac amorphous twin consistent with Twin Reversed Arterial Perfusion (TRAP) sequence.

**Discussion:**

Twin Reversed Arterial Perfusion (TRAP) sequence is a rare and severe complication of monochorionic twin pregnancies, characterized by reversed perfusion of a nonviable acardiac twin through vascular anastomoses from the pump twin, putting the latter at risk of cardiac failure. Diagnosis relies on prenatal imaging, especially ultrasound and Doppler studies, with fetal echocardiography and MRI aiding in risk assessment and intervention planning. Early fetal therapy techniques such as radiofrequency ablation or laser photocoagulation have significantly improved outcomes, although the optimal timing of intervention remains under discussion.

**Conclusion:**

This case underscores the complexity of TRAP sequence and the critical role of early ultrasound monitoring and intervention in improving outcomes for the pump twin.

## Highlights

• TRAP sequence is a rare complication of monochorionic twin pregnancies, often misdiagnosed due to its atypical presentation.
• This case mimicked a placenta bipartita, delaying the recognition of an acardiac twin.
• Prenatal ultrasound remains essential in identifying reversed arterial perfusion and guiding early intervention.
• Macroscopic and histologic findings confirmed the diagnosis of acardius amorphus.

## 1 Introduction

Twin reversed arterial perfusion (TRAP) sequence is a rare condition that complicates approximately 1–2 % of monochorionic twin pregnancies. It is characterized by the absence of a functioning heart in one twin, which receives its blood supply from the “pump” twin via arterio-arterial and veno-venous anastomoses [3]. This condition is diagnosed primarily through ultrasound, which can identify the reversed blood flow and associated complications such as polyhydramnios and cardiac failure in the “pump” twin [2]. Recent studies have shown that early intervention, including and laser photocoagulation, can significantly improve outcomes for the “pump” twin by reducing the risk of cardiac failure and premature birth [2]. We present a case of TRAP sequence diagnosed initially as a placenta bipartita. We aim to highlight the role of early antenatal screening. This work has been reported in line with the SCARE criteria. [1]

## 2 Case report

**Fig. 1:**
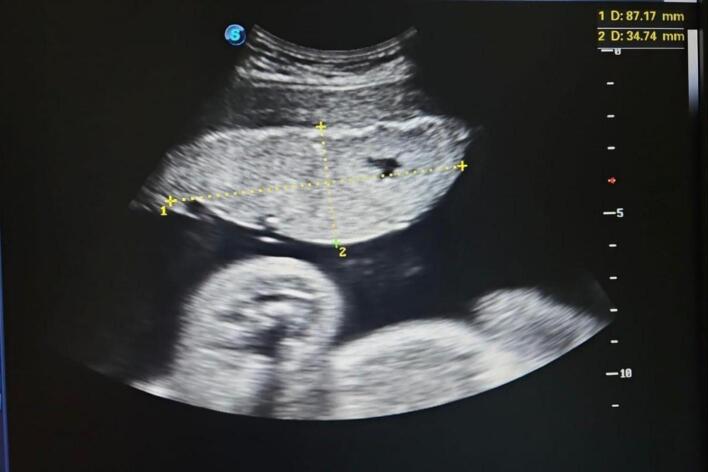
Ultrasound of bipartita placenta.

A 33-year-old Gravida 2 Para 1, with no significant medical history was referred to our hospital due to decreased fetal movements at term. The prenatal evaluation at admission was normal. Ultrasonography showed monofetal pregnancy estimated at 34 weeks with negative cardiac activity and a placenta bipartita (Fig. 1).

Two hours after admission, the patient's membranes ruptured spontaneously. The amniotic fluid was stained, and the cervix was dilated by about one finger. She was placed under antibiotic prophylaxis and hygiene measures. However, she developed a fever of 38.5 °C rectally, 9 h after the rupture of membranes.

**Fig. 2:**
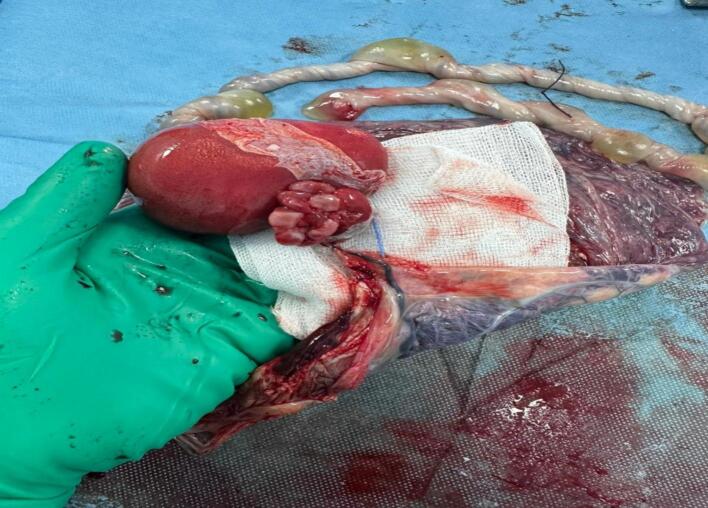
Acardiac twin.

**Fig. 3:**
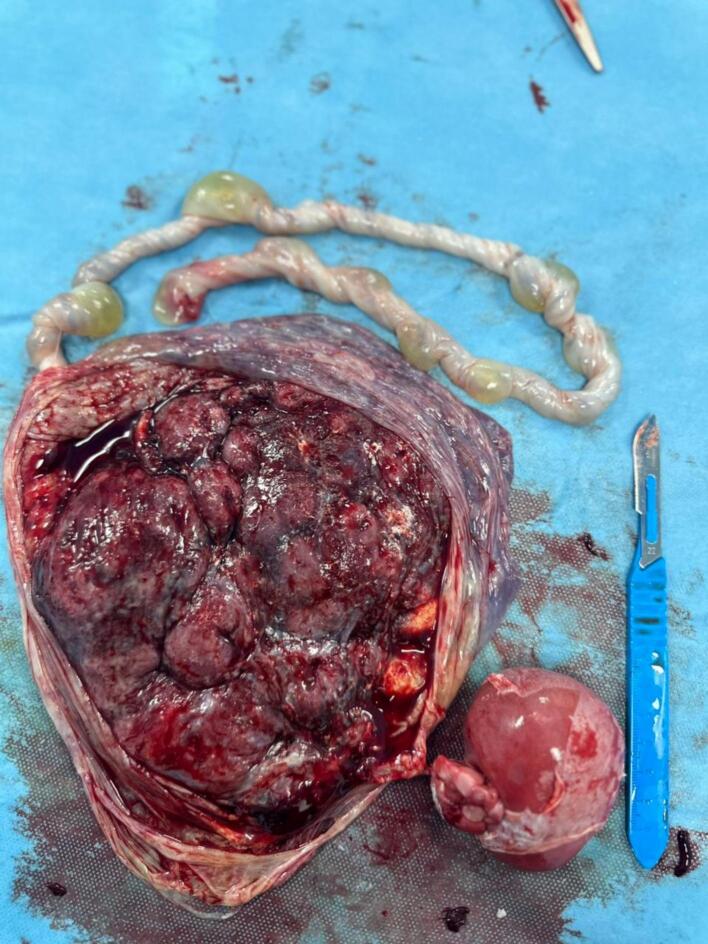
The placenta and the acardiac twin.

The patient was taken up for section cesarean section for chorioamnionitis due to an unfavorable Bishop score, which resulted in the extraction of a stillborn male baby weighing 2420 g with a mass measuring approximately 7 cm in its greatest diameter, suggesting an acardiac twin (Fig. 2). The placenta and a single amniotic sac contained both the fetus and the mass (Fig. 3).

**Fig. 4:**
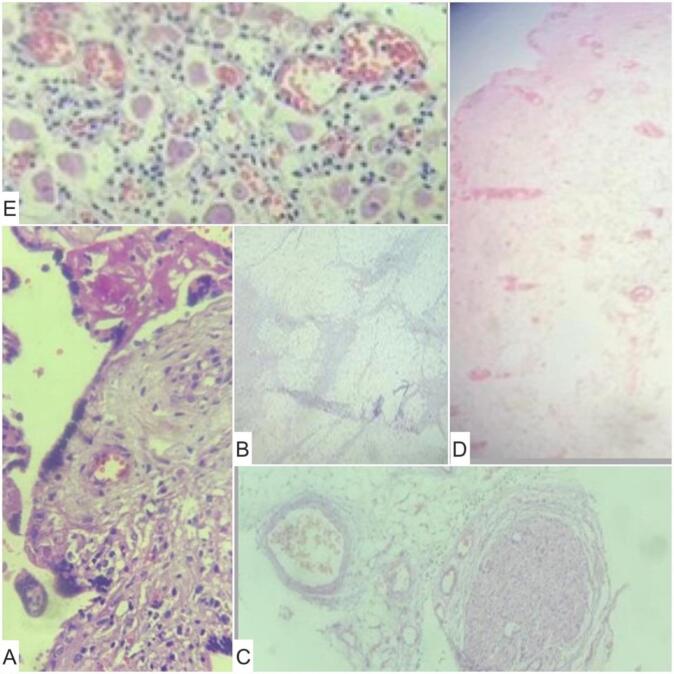
Microscopic examination. A: Chorionic villus with vascularized fibrous axis infiltrated by small lymphocytes and some histiocytes; B: lobules of adipose tissue made up of mature adipocytes; C: rare nerve fibers; D: sweat gland; E: mature ganglion cells.

The macroscopic examination revealed a placenta weighing 406 g and measuring 19x14x3 cm, attached to a mass connected by a few vessels. The mass measured 7 × 6 cm and appeared brownish, with a thin, whitish layer adherent to its surface and the microscopic showed a mass containing lobules of adipose tissue made up of mature adipocytes, sweat glands, rare nerve fibers, mature ganglion cells, calcified bone tissue, and undifferentiated mesenchymal tissue (Fig. 4). The appearance is suggestive of a TRAP or reverse arterial perfusion sequence with acardius amorphus.

Postpartum follow-up showed an uneventful maternal recovery, with no surgical or medical complications. The patient's physical condition returned to normal within a few weeks, and routine gynecological follow-up confirmed a satisfactory recovery. From the patient's perspective, she expressed relief that the cause of her condition was finally clarified, although she experienced psychological distress due to the unfavorable pregnancy outcome. She emphasized the importance of clear communication and support throughout her management. The patient benefited from continuous psychological support provided by the medical team, which helped her cope with the distressing news. Counseling sessions, combined with strong family support, played a crucial role in allowing her to gradually overcome the grief and regain emotional stability.

## 3 Discussion

Twin reversed arterial perfusion (TRAP) sequence is a rare but severe complication primarily affecting monochorionic twin pregnancies, where one twin acts as a “pump” for an acardiac twin [1]. This condition is characterized by vascular anastomoses on the placental surface, leading to perfusion of the acardiac twin with deoxygenated blood, which can cause significant malformations and put the pump twin at risk of cardiac failure [3]. Umbilical cord anomalies are seen in up to 97 % of cases. The most common cord anomaly is a single umbilical artery of the acardiac twin. Arterial flow, which is expected to return to the placenta, passes through the pump fetus directly to the receiver fetus [4].

Ultrasound plays a critical role in diagnosing TRAP (Twin Reversed Arterial Perfusion) sequence and identifying high-risk factors such as polyhydramnios and abnormal Doppler waveforms [5]. The differential diagnosis of TRAP includes fetal death in utero, fetus papyraceus, vanishing twin syndrome, and twin-to-twin transfusion syndrome, which must be carefully considered when evaluating a twin pregnancy with abnormal findings [6]. Furthermore, advances in fetal imaging have improved the detection of TRAP sequence. High-resolution ultrasound and magnetic resonance imaging (MRI) can provide detailed information about the anatomy of the acardiac twin and the extent of vascular anastomoses, aiding in the planning of interventions [7]. Additionally, the use of fetal echocardiography is crucial for monitoring cardiac function in the pump twin, allowing for timely intervention if signs of cardiac strain are detected [5].

Recent studies have highlighted the importance of early intervention in managing TRAP sequence. Techniques such as radiofrequency ablation (RFA) and laser photocoagulation are commonly used to interrupt blood flow to the acardiac twin, and reducing the risk of cardiac failure in the pump twin [2]. A study comparing RFA and laser photocoagulation found no significant difference in neonatal survival rates, suggesting that both methods are effective [8]. Radiofrequency ablation is a minimally invasive, percutaneous technique that can effectively obliterate blood supply to an acardiac twin to preserve and protect the pump twin. Intra-fetal radiofrequency ablation technique can be decided due to the Umbilical artery Doppler ultrasound study of the pump twin showed abnormal end-diastolic flow and increased in umbilical arterial systolic/diastolic ratio at the later gestational age of the pregnancy [9].

One such approach, bipolar cord coagulation combined with amniopatch and amnioinfusion, has been reported. This procedure reduces cardiac strain on the pump twin and increases the chance of survival, and may be considered when specialized fetal therapy centers are available [10].

The optimal timing of intervention remains a topic of debate, although early intervention, ideally in the first trimester, is increasingly recommended to improve outcomes for the pump twin [10].

In conclusion, managing twin pregnancies with an acardiac mass requires a multidisciplinary approach, including early ultrasound diagnosis and intervention to optimize outcomes for the pump twin. Recent advances in fetal interventions have significantly improved survival rates, emphasizing the need for continued research into optimal treatment strategies [3].

## 4 Conclusion

In conclusion, our case highlights the complexity of twin pregnancies with an acardiac mass and emphasizes the importance of regular ultrasound surveillance and early interventions to optimize outcomes for the “pump” twin. Future research should focus on improving diagnostic and therapeutic techniques for these rare but severe cases.

## Author contribution

Sofia MCHICHOU, Rim LAABOUDI, Chayma CHAKIR: performed surgery, paper writing and editing.

Samir BARGACH, Fatima Elhassouni: literature review, Supervision.

Rim LAABOUDI, Sofia MCHICHOU, Soundous AMINE: Manuscript editing, picture editing

## Consent for publication

Written informed consent was obtained from the patient to publish this case report and accompanying images. On request, a copy of the written consent is available for review by the Editor-in-Chief of this journal.

## Ethical approval

The case report is not containing any personal information. Case reports are exempt from ethical approval in our institute.

## Guarantor

Sofia MCHICHOU M.D.

## Research registration number

Not applicable.

## Funding

The authors declare no funding or grant support.

## Conflict of interest statement

The authors declare that they have no competing interests relevant to the content of this article.

## References

[1] Kerwan A., Al-Jabir A., Mathew G., Sohrabi C., Rashid R., Franchi T., Nicola M., Agha M., Agha R.A. (2025). Revised Surgical CAse REport (SCARE) guideline: an update for the age of Artificial Intelligence. Prem. J. Sci..

[2] Trabelsi W., Abdennadher G., Louati W., Kallel W., Khemiri H., Guermazi S., Rekik S. (2004). Grossesse gémellaire compliquée d'un fœtus acardiaque. J. Int. Med..

[3] Lewi L., Valencia C., Gonzalez E., Deprest J., Nicolaides K.H. (2010). The diagnosis and management of twin reversed arterial perfusion sequence. Am. J. Obstet. Gynecol..

[4] Dudy L., Kumar S. (2022).

[5] (2022). Twin reversed arterial perfusion sequence: prenatal diagnosis. J. Matern.-Fetal Neonatal Med..

[6] Dhanju Gurinder, Breddam Alli (2020). Twin reversed arterial perfusion (TRAP) sequence: a case report and a brief literature review. Radiol. Case Rep..

[7] (2023). Twin reversed arterial perfusion sequence: current treatment options. Int. J. Women’s Health.

[8] (2024). Cord occlusion in twin reversed arterial perfusion sequence. Fetal Diagn. Ther..

[9] Chan K.-S., Chuang Y.-C., Lin T.-Y., Shaw S.W. (2021). A Taiwan’s experience: a case report and review of literature of successful early intrauterine treatment with radiofrequency ablation in twin reversed arterial perfusion (TRAP) sequence. J. Formos. Med. Assoc..

[10] Aldiansyah D., Lubis M.P., Handayani D., Asroel E.M., Barus M.N.G., Lubis B.M. (2022). Twin reversed arterial perfusion sequence managed by bipolar cord coagulation and amniopatch: case report. Int. J. Surg. Case Rep..

